# Associations between women’s empowerment, care seeking, and quality of malaria care for children: A cross-sectional analysis of demographic and health surveys in 16 sub-Saharan African countries

**DOI:** 10.7189/jogh.12.04025

**Published:** 2022-03-19

**Authors:** Todd P Lewis, Youssoupha Ndiaye, Fatuma Manzi, Margaret E Kruk

**Affiliations:** 1 Department of Global Health and Population, Harvard T.H. Chan School of Public Health, MA, USA; 2 Division of Planning, Research and Statistics, Ministry of Health and Social Action, Dakar, Senegal; 3 Department of Health Systems, Impact Evaluation and Policy, Ifakara Health Institute, Dar es Salaam, Tanzania

## Abstract

**Background:**

Fever and malaria are highly prevalent among children under five across sub-Saharan Africa, but utilization and quality of care for febrile illness remain insufficient. Many studies examine socioeconomic and demographic determinants of care seeking; however, few assess how women’s empowerment influences care seeking and quality. We examine associations of women’s empowerment with: a) care utilization for children with fever and malaria and b) the quality of that care in 16 sub-Saharan African countries.

**Methods:**

This cross-sectional study used data from Demographic and Health Surveys conducted between 2010 and 2018. We constructed indices for economic, educational, sociocultural, and health-related empowerment and calculated the proportion of children with fever and malaria who sought care and received a range of recommended clinical actions. We used multivariable Poisson hurdle models to assess associations between empowerment, utilization, and number of components of quality care, controlling for socioeconomic and demographic factors.

**Results:**

Our sample consisted of 25 871 febrile children, 4731 of whom had malaria diagnosed by rapid diagnostic test. Empowerment among mothers of children with fever was 0.50 (interquartile range, 0.38-0.63). In both the fever and malaria groups, over 30% of children were not taken for care. Among care seekers, febrile children received on average 0.47 (SD = 0.37) of components of quality care, and children with malaria received 0.38 (SD = 0.34). Multidimensional women’s empowerment was significantly associated with care seeking and quality among febrile children, and with quality among children with malaria. Associations persisted after adjustment for socioeconomic and demographic characteristics.

**Conclusions:**

Results demonstrate substantial gaps in women’s empowerment and poor utilization and quality of care for fever and malaria among children. Increased women’s empowerment is associated with seeking care and, separately, obtaining high-quality care. To improve health outcomes, consideration of how empowering women can promote care seeking and extract quality from the health system is warranted.

An estimated six million children under five years old die each year worldwide and over half of these deaths occur in sub-Saharan Africa [1]. Malaria, the third leading cause of mortality in children under five years of age, resulted in 7% of these deaths [1]. However, some progress has been made in recent decades with the availability of low-cost vector control strategies and effective preventive therapies: global malaria mortality decreased nearly 30% between 2010 and 2017 [2]. Despite these gains, the high burden of malaria persists, especially among children under five who accounted for 61% of malaria deaths worldwide in 2017 [2].

Averting severe malaria and related mortality requires prompt diagnosis and treatment, though utilization of high-quality care for fever and malaria remains low [2,3]. National household surveys conducted in 19 sub-Saharan African countries between 2015 and 2017 indicate that a median of 52% of febrile children were taken to any type of trained medical provider for care [2]. Febrile children who seek care should receive a malaria rapid diagnostic test (RDT) or microscopy to confirm malaria diagnosis, but many do not [4,5]. Children with confirmed malaria should be treated with appropriate antimalarial drugs, such as artemisinin-based combination therapy (ACT) [6]. Despite wide availability and efficacy, appropriate use of ACTs among febrile children is low [5,7]. Further, children with negative RDT results frequently receive inappropriate treatment, including unindicated antibiotics or antimalarials [4,5].

In recent years, research on the determinants of care seeking and quality of care for children has expanded to include dimensions of women’s empowerment, defined as “the process of change wherein an individual with prior inability to choose has the access and freedom to make choices” [8,9]. Existing literature, largely qualitative, provides evidence that factors related to empowerment such as household structures and power dynamics have a substantial impact on utilization of care, including for malaria [10-12]. However, this literature is largely limited to utilization decisions, with little focus on how empowerment may influence subsequent quality of care. Further, few studies assess multiple dimensions of women’s empowerment, such as decision-making power, interpersonal autonomy, or social status, that may be associated with both utilization and quality of care for sick children.

We used survey data from 16 countries in sub-Saharan Africa to examine the relationship between women’s empowerment and care for children with fever and malaria. We constructed four indices of women’s empowerment to assess the extent to which empowerment determines care seeking and receipt of high-quality care for sick children. Results can be used to understand how multidimensional empowerment, beyond the usual measures of education and wealth, may influence care quality. This insight may inform potential interventions to raise people’s demand for quality and reduce malaria mortality in sub-Saharan Africa.

## METHODS

### Study sample

Data for each country were obtained from the Demographic and Health Surveys (DHS), which conducts nationally-representative household surveys of population, health, and nutrition [13]. The Household Questionnaire collects information on basic household characteristics, such as household wealth. The Woman’s Questionnaire, which surveys women ages 15 to 49 years, includes a limited set of indicators regarding women’s status and empowerment. Since 2000, DHS surveys have included indicators regarding malaria prevention and treatment and testing for malaria parasites using rapid diagnostic tests for children under age five. Sampling strategies and methods have been previously described [14].

We used the most recent survey available in each country in the last ten years for countries that included malaria biomarker testing in their survey, including the following 16 countries: Angola, 2015-16; Benin, 2017-18; Burkina Faso, 2010; Burundi, 2016-17; Côte d’Ivoire, 2011-12; Democratic Republic of the Congo, 2013-14; Gambia, 2013; Ghana, 2014; Guinea, 2012; Mali, 2012-13; Mozambique, 2011; Rwanda, 2014-15; Senegal, 2017; Tanzania, 2015-16; Togo, 2013-14; and Uganda, 2016.

We analyzed receipt of care among two groups of children under five years old: 1) children with fever reported in the last two weeks and 2) children with fever reported in the last two weeks who had malaria diagnosed by RDT. To reduce recall bias from mothers’ self-reports, we included the youngest child under five only.

### Outcome definition and assessment

Using indicators available in the DHS surveys, we developed two primary outcomes to assess the number of components of high-quality care obtained by children who sought treatment for fever (four items) and malaria (six items). Both outcomes included the following components: 1) whether the mother sought any advice or treatment for her child; 2) whether the mother sought care at a formal facility or provider; 3) whether the child had blood taken from the finger or heel for testing; and 4) whether the child did not receive inappropriate treatment, defined as receipt of a contraindicated or unrecommended medication according to country guidelines, such as an outdated malarial.

The outcome for children with malaria included two additional components: 5) whether the child received timely treatment, defined as the child beginning treatment on the same day or next day after the fever started; and 6) whether the child received correct treatment, defined as receipt of an appropriate antimalarial according to a country’s treatment guidelines. The final outcome is a count of items ranging from zero to four among the sample of children with fever and zero to six in the subsample of children with diagnosed malaria. A score of zero indicates no care for fever was sought, while four or six indicates care was sought and all components of high-quality care were obtained among children with fever and malaria respectively.

### Covariates

**Figure 1:**
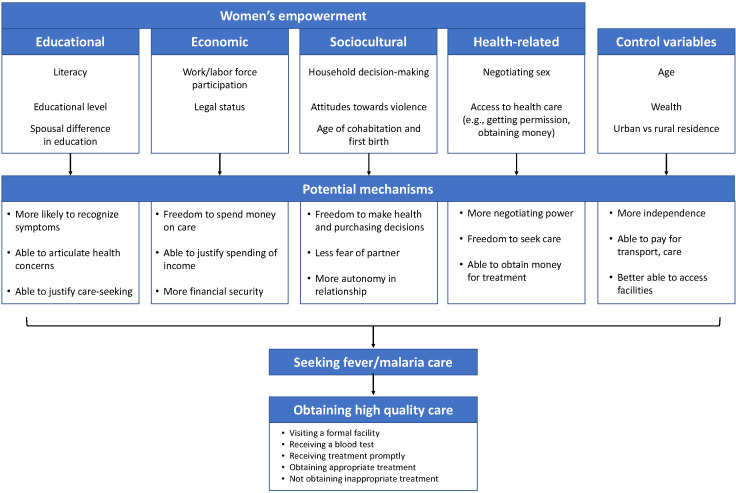
Conceptual model of women’s empowerment and care seeking for children with fever and malaria.

We created a conceptual model of women’s empowerment based on validated models that use women’s status indicators available in the DHS (**Figure 1**, Figure S1 in the **Online Supplementary Document**) [8,15-17]. We modified the conceptual framework to include 25 indicators of empowerment most likely to be associated with seeking care and obtaining high-quality care for a child with fever or malaria. Our model includes four dimensions of empowerment: educational, economic, sociocultural, and health-related empowerment. Each dimension is composed of multiple domains, such as labor and workforce participation, household decision-making, and attitude towards violence, that categorize specific empowerment indicators. Indicators within each domain are binary, with a one indicating greater empowerment (Table S1 in the **Online Supplementary Document**). A woman’s empowerment score in a given dimension is calculated as the mean proportion of empowerment indicators she experienced across domains within each dimension of empowerment. The resulting dimension score ranges from zero to one with a higher score corresponding to greater empowerment.

In addition to empowerment, our models include mother, child, and household characteristics that may influence care seeking and obtaining high-quality care, such as child sex, maternal and child age, type of place of residence (urban vs rural), and household wealth quintile.

### Statistical analysis

First, we calculated basic descriptive characteristics of mothers and their children with fever or malaria. We calculated empowerment scores overall in the sample, by each dimension of empowerment, and by country. To assess utilization and quality, we calculated the proportion of children who sought care and received each component of high-quality care. We compared empowerment scores in each dimension among children who did and did not obtain each component of care for fever or malaria, and tested significance of differences.

Second, we constructed multivariable hurdle regression models to test the association between empowerment and utilization and quality of care (additional description in the **Online Supplementary Document**). In the first stage, we used logistic regression to determine the odds of care seeking. In the second stage, we used a zero-truncated Poisson model to determine the count of recommended care items a child received, expressed as incidence rate ratios. The first model tested associations with the four-item outcome among children with fever and the second model tested associations with the six-item outcome among children with malaria. Both models controlled for mother, child, household characteristics, and country fixed effects.

Finally, we tested associations between empowerment and each component of care in separate models, and associations between overall empowerment averaged across dimensions and both the four-item and six-item outcomes. We also included an interaction term in this model to assess whether wealth modified the relationship between empowerment and either outcome. To test whether the associations held in areas with greater disease prevalence and health system familiarity with malaria, we estimated our models among countries where malaria transmission occurs year-round separately from countries with seasonal transmission. All statistical analyses were carried out using Stata version 14.2 (Stata-Corp, College Station, TX, USA).

### Ethical approval

The original survey implementers obtained ethical approvals for data collection; the institutional human research protection program deemed this analysis based on deidentified data in the public domain as exempt from human subjects review.

## RESULTS

The DHS surveys included 111 339 mothers and 159 717 children under age five across the 16 countries of interest, among whom 108 531 children were the youngest and had mothers interviewed regarding empowerment. The analytic samples included 25 871 children who had fever in the last two weeks and a subset of 4731 children who had fever in the last two weeks and malaria diagnosed by RDT.

**Table 1** describes characteristics of mothers and children in both analytic samples across all 16 countries. A majority of the children were under age three in both samples. Most mothers were between 20 to 34 years old. At least one quarter of mothers and children in both samples were in the lowest household wealth quintile in their country, with the majority in both samples living in the bottom three wealth quintiles.

**Table 1:** Description of mothers, their children with fever, and their children  diagnosed with malaria*†

	Characteristics of mothers and their children with fever (N = 25 871)		Characteristics of mothers and their children with malaria (N = 4731)	
**Variable**	**N**	**%**	**N**	**%**
Child sex:				
Female	12 564	49	2293	48
Child age (years):				
<1	7034	27	674	14
1	8495	33	1677	35
2	5560	21	1314	28
3	2963	11	650	14
4	1819	7	416	9
Woman age (years):				
15-19	1777	7	287	6
20-24	5808	22	1004	21
25-29	6577	25	1166	25
30-34	5302	20	941	20
35-39	3742	14	716	15
40-44	2013	8	449	9
45-49	652	3	168	4
Household wealth:				
Poorest	6573	25	1477	31
Poorer	5862	23	1252	26
Middle	5232	20	968	20
Richer	4709	18	757	16
Richest	3495	14	277	6
Rural/non-rural:				
Rural	19 267	74	4072	86
Country:				
Angola	1017	4	114	2
Benin	892	3	98	2
Burkina Faso	2480	10	850	18
Burundi	3369	13	765	16
Côte d'Ivoire	1293	5	266	6
DRC	3494	14	641	14
Gambia	782	3	6	<1%
Ghana	653	3	167	4
Guinea	1343	5	360	8
Mali	606	2	161	3
Mozambique	1013	4	194	4
Rwanda	1135	4	73	2
Senegal	1978	8	39	1
Tanzania	1274	5	207	4
Togo	1096	4	242	5
Uganda	3446	13	548	12
**Outcome: Care for fever and malaria**				
Sought any treatment:	17 756	69	3225	68
	**Mean**	**SD**	**Mean**	**SD**
Proportion of care items received (out of 4 for fever; out of 6 for malaria)	0.47	0.37	0.38	0.34

DRC – Democratic Republic of the Congo

*Household wealth is defined by DHS as a country-specific composite measure of a household's cumulative living standard based on ownership of selected assets.

†Care items received for children with fever include whether the mother/child sought treatment at a formal facility, had blood taken from the finger or heel for testing, and began treatment for fever/malaria the same or next day. Care items received for children with malaria include those for children with fever as well as whether the child received appropriate treatment and did not receive inappropriate treatment.

**Figure 2:**
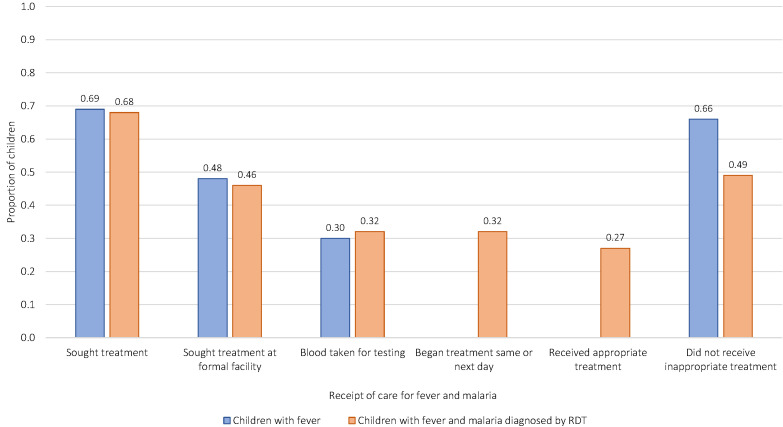
Proportion of children obtaining each component of care for fever (N = 25871) or malaria (N = 4731). “Sought treatment at formal facility” is defined as seeking care at a formal facility or provider, such as a government health center, rather than an informal provider such as a traditional practitioner or marketplace. “Received appropriate treatment” is defined as receipt of an antimalarial deemed appropriate by each country’s national malaria treatment guidelines for either uncomplicated or severe malaria (typically an artemisinin-based combination therapy). “Did not receive inappropriate treatment” is defined as avoidance of a contraindicated medication or an unrecommended drug for a positive malaria diagnosis, a negative malaria diagnosis, or fever but unknown malaria according to country-specific guidelines.

Levels of care seeking and receipt of high-quality care were low in the sample and varied across countries (**Figure 2****,** Figure S2 in the **Online Supplementary Document**). On average, febrile children received 0.47 (standard deviation (SD) = 0.37) of care items, while children with malaria received 0.38 (SD = 0.34). This means that children received approximately one additional clinical action beyond seeking care on average in both samples. Care seeking was low with only 69% of mothers of febrile children and 68% of mothers of children with malaria seeking any treatment. Of these, 48% of mothers of febrile children and 46% of mothers of children with malaria sought treatment at a formal facility, and only 30% of febrile children and 32% of children with malaria had blood taken for testing. A small majority of children avoided inappropriate treatment for fever (66%), while just under half of children with malaria (49%) avoided inappropriate treatment. Only 32% of children with malaria began treatment the same or next day as onset of fever and only 27% of children with malaria received an appropriate antimalarial.

**Figure 3:**
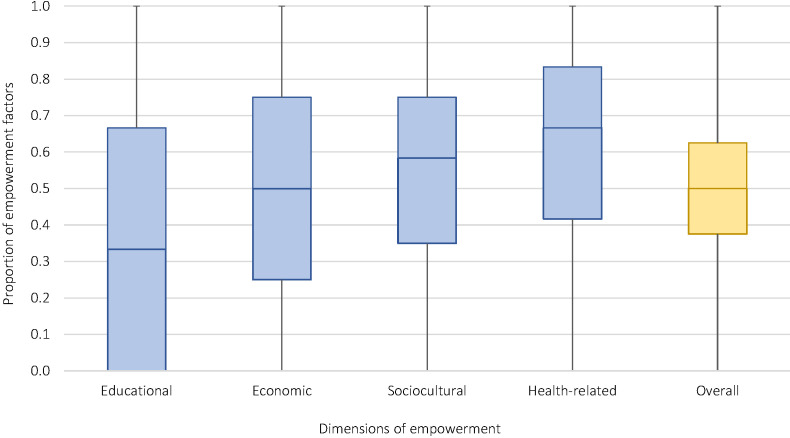
Proportion of empowerment factors experienced by mothers of children with fever by dimension (N = 25871). Educational empowerment is an average of the proportion of empowerment factors a woman experiences in three domains: literacy, educational level, and spousal difference in education (three indicators total). Economic empowerment is an average of the proportion of empowerment factors a woman experiences in two domains: work/labor force participation and legal status (six indicators total). Sociocultural empowerment is an average of the proportion of empowerment factors a woman experiences in three domains: household decision-making, attitudes towards violence, and life course (11 indicators total). Health-related empowerment is an average of the proportion of empowerment factors a woman experiences in two domains: negotiating sex and access to health care (five indicators total).

**Figure 4:**
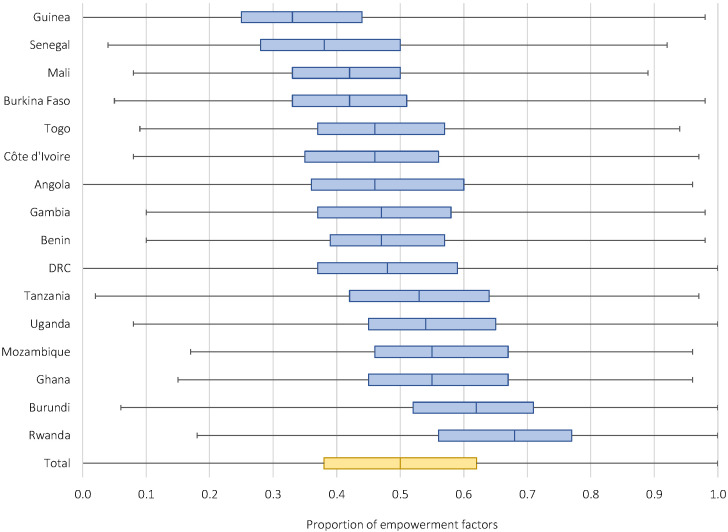
Proportion of empowerment factors experienced by mothers of children with fever by country (N = 25 871). DRC – Democratic Republic of the Congo. Empowerment is calculated as an average of the proportion of empowerment factors a woman experiences in four empowerment dimensions: educational, economic, sociocultural, and health-related (25 indicators total).

Women in the sample of children with fever had a median empowerment score of 0.50 (IQR = 0.38-0.63) out of a 1.00 maximum index score, indicating half of women experienced 50% or fewer of the measured components of empowerment (**Figure 3**). Scores varied by country (**Figure 4****,** Table S2 in the **Online Supplementary Document**). Educational empowerment was lowest, with a median empowerment score of only 0.33 (IQR = 0-0.66), while health-related empowerment was highest, with a score of 67% (median, 0.67; IQR = 0.42-0.83). Overall women’s empowerment in the sample of children with malaria diagnosed by RDT was similar but slightly lower than that in the sample of children with fever, with a median empowerment score of 0.46 (IQR = 0.35-0.57) (data not shown). Empowerment also varied substantially by country, ranging from 33% (median, 0.33; IQR = 0.24-0.44) in Guinea to 68% (median, 0.68; IQR = 0.56-0.77) in Rwanda (Table S2 in the **Online Supplementary Document**). Bivariable associations suggest that children who were taken to care and obtained high-quality care had mothers who were equally or more empowered in every dimension compared to children who were not (Table S3 in the **Online Supplementary Document**).

**Table 2** presents the results of the fully-adjusted hurdle regression models focusing on the effect of empowerment on seeking care and obtaining high-quality care among children with fever and malaria. Among children with fever in the last two weeks, educational empowerment, sociocultural empowerment, and health-related empowerment were significant predictors of seeking any care for fever, though effect sizes were small. Health-related empowerment had the largest effect: a one-unit increase (from no empowerment to complete empowerment) in health-related empowerment was associated with a 32% increase in the odds of seeking care (95% confidence interval (CI) = 1.08, 1.62). Wealth and maternal age were also significantly associated with care seeking. Mothers in the country’s wealthiest quintile had a 58% increase in the odds of seeking care as compared to those in the poorest.

**Table 2:** Multivariable hurdle regressions on care seeking and receipt of quality care for children with fever (N = 25 871) and malaria (N = 4731)*†‡

	Children with fever in the last two weeks				Children with malaria diagnosed by RDT			
	**Logistic part: Sought care (yes or no)**		**Poisson part: Number of care items (if care was sought)**		**Logistic part: Sought care (yes or no)**		**Poisson part: Number of care items (if care was sought)**	
**Variable**	**AOR**	**95% CI**	**AIRR**	**95% CI**	**AOR**	**95% CI**	**AIRR**	**95% CI**
Educational empowerment	**1.11**	**(1.037-1.187)**	1.01	(0.991-1.031)	0.90	(0.737-1.105)	1.03	(0.984-1.084)
Economic empowerment	1.14	(0.995-1.312)	1.03	(0.998-1.070)	1.03	(0.726-1.448)	1.05	(0.981-1.127)
Sociocultural empowerment	**1.22**	**(1.072-1.379)**	**1.05**	**(1.012-1.084)**	1.00	(0.810-1.243)	**1.10**	**(1.021-1.176)**
Health-related empowerment	**1.32**	**(1.079-1.621)**	**1.04**	**(1.003-1.080)**	1.14	(0.895-1.454)	**1.09**	**(1.022-1.165)**
Female	1.01	(0.958-1.068)	0.99	(0.986-1.003)	0.97	(0.863-1.099)	0.99	(0.972-1.018)
Child age	1.01	(0.985-1.045)	1.00	(0.990-1.002)	**0.92**	**(0.865-0.979)**	1.01	(0.993-1.021)
Mother age	**0.99**	**(0.987-0.996)**	1.00	(0.998-1.000)	0.99	(0.982-1.002)	1.00	(0.998-1.002)
Rural	0.92	(0.799-1.050)	0.98	(0.951-1.011)	0.96	(0.738-1.261)	0.97	(0.910-1.024)
Wealth (ref: Poorest quintile):								
Second quintile	1.06	(0.925-1.204)	1.00	(0.967-1.037)	1.07	(0.937-1.217)	1.01	(0.952-1.064)
Third quintile	1.15	(0.975-1.363)	1.00	(0.965-1.043)	1.17	(0.893-1.528)	1.02	(0.972-1.080)
Fourth quintile	**1.25**	**(1.023-1.520)**	1.01	(0.964-1.049)	**1.36**	**(1.068-1.729)**	1.05	(0.974-1.125)
Richest quintile	**1.58**	**(1.179-2.129)**	1.01	(0.963-1.057)	**2.26**	**(1.646-3.099)**	1.09	(0.998-1.181)
Observations	25871		17 756		4731		3225	

AOR – adjusted odds ratio, AIRR – adjusted incidence rate ratio, CI – confidence interval

*Educational empowerment is an average of the proportion of empowerment factors a woman experiences in three domains: literacy, educational level, and spousal difference in education (three indicators total). Economic empowerment is an average of the proportion of empowerment factors a woman experiences in two domains: work/labor force participation and legal status (six indicators total). Sociocultural empowerment is an average of the proportion of empowerment factors a woman experiences in three domains: household decision-making, attitudes towards violence, and life course (11 indicators total). Health-related empowerment is an average of the proportion of empowerment factors a woman experiences in two domains: negotiating sex and access to health care (five indicators total).

†Care items for children with fever include seeking care at a formal facility, having blood taken, and not receiving inappropriate treatment. Care items for children with malaria include seeking care at a formal facility, having blood taken, beginning treatment the same or next day, receiving appropriate treatment, and not receiving inappropriate treatment.

‡Estimates were obtained using Poisson hurdle regression clustered at the country level. All models included country fixed effects. Bold values are statistically significant at the *P* = 0.05 level.

Sociocultural and health-related empowerment were both associated with increases in the count of high-quality care items among febrile children. A doubling of the index of sociocultural empowerment was associated with a 5% increase in the rate of care items received (95% CI = 1.01-1.08), while the same increase in health-related empowerment was associated with a 4% increase in the rate of items received (95% CI = 1.00-1.08). If mothers of children with fever were fully empowered across each dimension, predictions based on the hurdle regression model suggest that children with fever would receive on average two of the four components of high-quality fever care (data not shown). No socioeconomic or demographic characteristics predicted meaningful change in the count of care items received by febrile children.

In the sample of children with diagnosed malaria, mothers of older children were significantly less likely to seek care for malaria (AOR = 0.92, 95% CI = 0.87-0.98), and mothers in the fourth and fifth wealthiest quintiles in their countries were highly associated with seeking treatment: on average, mothers in the richest quintile had 2.26 times the odds of seeking care for their children as compared to mothers in the poorest quintile (95% CI = 1.65-3.10).

As among febrile children, sociocultural empowerment and health-related empowerment were significantly associated with the count of high-quality care items received among children with malaria. A doubling of the sociocultural empowerment index was associated with a 10% increase in the rate of care items received (95% CI = 1.02-1.18), while the same increase in health-related empowerment was associated with a 9% increase (95% CI = 1.02-1.17). If mothers of children with malaria were fully empowered across each dimension, our models predict children with malaria would receive on average approximately three of the six components of high-quality malaria care (data not shown). In contrast, the effects of socioeconomic and demographic covariates were small in magnitude and not significant.

When testing associations using an overall empowerment score across dimensions, results are similar to our main models: Overall empowerment is highly associated with seeking care (AOR = 2.01, 95% CI = 1.57, 2.56) and obtaining high-quality care (AIRR = 1.13, 95% CI = 1.08, 1.18) among children with fever; among children with malaria, overall empowerment is not a significant predictor of seeking care, but is associated with a 29% increase in the rate of care items received (95% CI = 1.15-1.45) (Table S4 in the **Online Supplementary Document**). Logistic regressions for each component of care are also similar, though resulting coefficients are slightly larger in magnitude (Tables S5 and S6 in the **Online Supplementary Document**). When using zero-inflated Poisson regressions, our findings are very similar to those from the hurdle model (Table S7 in the **Online Supplementary Document**). Results of models that included an interaction between wealth and empowerment were also similar to those from our main models, though we find small but statistically significant decreases in the relationship between empowerment and quality received among children with malaria who have mothers in the third (*P* = 0.006), fourth (*P* < 0.001), and fifth (*P* = 0.015) wealthiest quintiles (results not presented). When examining results in countries with year-round vs highly seasonal malaria transmission, empowerment remained a significant predictor of utilization and quality in both country groups.

## DISCUSSION

We assessed utilization and quality of care for children with fever and malaria in 16 sub-Saharan African countries and found that on average only 69% of children with fever and 68% of children with malaria were taken for care. Among those who did seek care, children with fever received on average 0.47 (SD = 0.37) of care items, while children with malaria received only 0.38 (SD = 0.34). Consistent with prior studies, we found that multidimensional women’s empowerment is low across dimensions and countries [18-21]. However, results suggest that empowered mothers are more likely to seek care for their children and to obtain high-quality care. Though effect sizes were modest, these results persisted after adjustment for socioeconomic and demographic characteristics. This study benefits from the use of large, nationally-representative samples with parasitological diagnosis of malaria.

Our results show that sociocultural empowerment and health-related empowerment, which consist of a mother’s interpersonal autonomy, decision-making power, and health-seeking independence, influence a mother’s ability to seek care for her sick child [10,22-24]. Once care has been sought, empowerment is predictive of receipt of higher quality care for both fever and malaria. This relationship may reflect an empowered mother’s ability to extract quality care by identifying better facilities or negotiating higher quality from providers irrespective of her demographic profile or socioeconomic status. This is particularly striking given mothers may not know all elements of quality care and supply side constraints may limit receipt of both diagnostic tests and appropriate treatment with ACTs [5,25]. While relatively small in magnitude, these gains could result in receipt of diagnostic confirmation of malaria or an appropriate antimalarial drug, which could have dramatic effects on reducing avertable mortality [2,26]. Empowerment of women is commonly considered an important strategy for reducing gender inequity and fostering good health [8,9,16]. While quality of care is largely determined by the health system, our results align with past studies showing that more empowered patients or caregivers may be able to identify better quality clinics and negotiate good quality services from providers [27-30]. In particular, decision-making power, control of resources, and intra-household relationships have been shown to influence care seeking and overall child health [10,22,23,31,32].

Our results also show that the relationship between empowerment and care seeking and quality varies by the severity of illness. Empowerment was significantly associated with care seeking among children with fever, but not among children with malaria. It may be that mothers of young children with malaria, who are typically very visibly ill, seek out a trained medical professional irrespective of empowerment. Wealth may be the primary limiting factor for these very ill children: women in the richest wealth quintile in their country have over twice the odds of seeking care for a child with malaria as compared to those in the poorest quintile [33,34]. While empowerment was not a significant predictor of care seeking for children with malaria, sociocultural and health-related empowerment were both associated with receipt of high-quality care. These results suggest that empowerment, while not a critical factor in care seeking for severely ill children, may still play an important role in the extraction of high-quality care from the health system.

In contrast, educational empowerment, sociocultural empowerment, and health-related empowerment were all significantly associated with care seeking for fever. Mothers of febrile children, for whom illness may be harder to detect, may only seek care when they are sufficiently knowledgeable about symptoms or able to negotiate care seeking with their partners. These relationships reflect the important role of empowered mothers in appropriately identifying illness and advocating for necessary care. However, it is important to recognize that households may have multiple decisionmakers who may influence care seeking, but are less likely to inform subsequent quality care [10,12].

The observed associations between empowerment and both care seeking and quality were significant even after adjusting for commonly used sociodemographic characteristics. These socioeconomic and demographic factors, including household wealth, child age, and place of residence, were not significantly associated with receipt of quality care conditional on having sought care for fever [35,36]. While economic empowerment, which includes factors such as workforce participation and home or land ownership, was not a significant determinant of care seeking or quality in either sample, wealthier mothers were more likely to take their ill child for treatment as found elsewhere [3,36]. Maternal and child age also influenced care seeking: Among children with fever, older mothers were less likely to seek care for their children, and among children with malaria, older children were less likely to be taken for care; other sociodemographic characteristics such as child sex or place of residence, typically strong determinants of care seeking, were not significant in our models.

Past studies have explored the determinants of low utilization of care for febrile children, in which socioeconomic and demographic variables are prominent. Wealthier households, those living in urban areas, and women with secondary or higher education have been shown to be more likely to seek appropriate care [33,37]. Younger children are also more likely to be brought to care [37]. Building upon these findings, our results suggest that while sociodemographic factors influence care seeking behavior, maternal empowerment is a stronger predictor of the subsequent quality of care received.

### Limitations

This study is subject to some limitations. Care-seeking data are based on a mother’s self-report of the last two weeks and may suffer from recall bias [38]. Associations may also be influenced by delays in care seeking, as some mothers may intend to seek care for their children but have not yet done so by the time of the survey. In addition, children with diagnosed malaria who received appropriate treatment may have cleared the parasite prior to the survey. These children would be coded as having received antimalarials inappropriately given the negative test result, though the treatment was in fact appropriate. Duration of positivity is highly variable by RDT type and other factors, though persistent positivity is more common among children and those treated with ACT which may limit bias [39]. We are careful in interpreting the effects of individual empowerment components, as our models may lack power to sufficiently distinguish between them. There may also be residual confounding in regression estimates from unobserved variables such as maternal health status. As this is a cross-sectional study, we cannot infer causation between empowerment and utilization or quality. Finally, measurement of empowerment is limited to DHS indicators, though other useful frameworks exist [40,41]. These indicators are constructed and applied in different ways, which can make comparisons challenging. In particular, this analysis would benefit from measures of empowerment that extend beyond the individual level, such as indicators of community empowerment or local governance, though such measures were not available in the data set. However, this study aimed to use a readily available set of indicators comparable across countries. Given our limited knowledge of how indicators beyond socioeconomic and demographic characteristics influence receipt of care, the use of these indicators remains an important contribution.

## CONCLUSIONS

This study demonstrates that women’s empowerment is associated with both care seeking and quality of care for sick children and that it may be more a more important predictor than education and social position alone in extracting good quality care. Therefore, consideration of a broader array of determinants of care seeking and obtaining high-quality care for sick children is warranted. However, additional research is needed to develop rich insight into the mechanisms that enable empowered mothers to obtain higher quality care. In particular, rigorous evaluation of interventions to empower women and the effect on subsequent quality received can inform policy efforts to ignite demand for high-quality care among the population.

We found that empowerment was low across countries, and thus promoting women’s agency in economic, health, and family decisions, in addition to important intrinsic merits, may be a fruitful approach for improving health systems and promoting child survival. Any such efforts must consider local needs, expectations, malaria transmission dynamics, and health system features in each country context to effectively improve care seeking and quality of care across sub-Saharan Africa.

## Additional material


Online Supplementary Document
