# Supplementary material for: Associations between women’s empowerment, care seeking, and quality of malaria care for children: A cross-sectional analysis of demographic and health surveys in 16 sub-Saharan African countries
Source: J Glob Health. 2022 Mar 19;12:04025. doi: 10.7189/jogh.12.04025 (PMC8932460; doi:10.7189/jogh.12.04025)
Supplement: Online Supplementary Document [file jogh-12-04025-s001.pdf]

Supplementary materials

Additional description of study methods

Model description

Hurdle regression is a two-equation model for count outcomes with excess zeros. The first equation determines the likelihood of a binary outcome and the second equation examines the positive count of outcomes. Hurdle regression allows separate modelling of both processes and accounts for excess zeros in the dependent variables (31% of children with fever and 32% of children with malaria in the sample did not seek care).

To select our model, we compared the goodness of fit of a Poisson model, a Poisson hurdle model, and a negative binomial hurdle model, and found evidence supporting use of a two-equation Poisson model. We also found the hurdle model provides similar goodness of fit to a zero-inflated Poisson (ZIP) model that models individuals not "at risk" of the outcome and those "at risk" but with a zero count. Results from zero-inflated Poisson regressions are very similar to those from the hurdle model (Appendix table 7). As children who did not seek care are unable to receive subsequent components of care, we selected a Poisson hurdle as our final model.

Figure S1. Full conceptual model of women's empowerment and care seeking for children with fever and malaria.

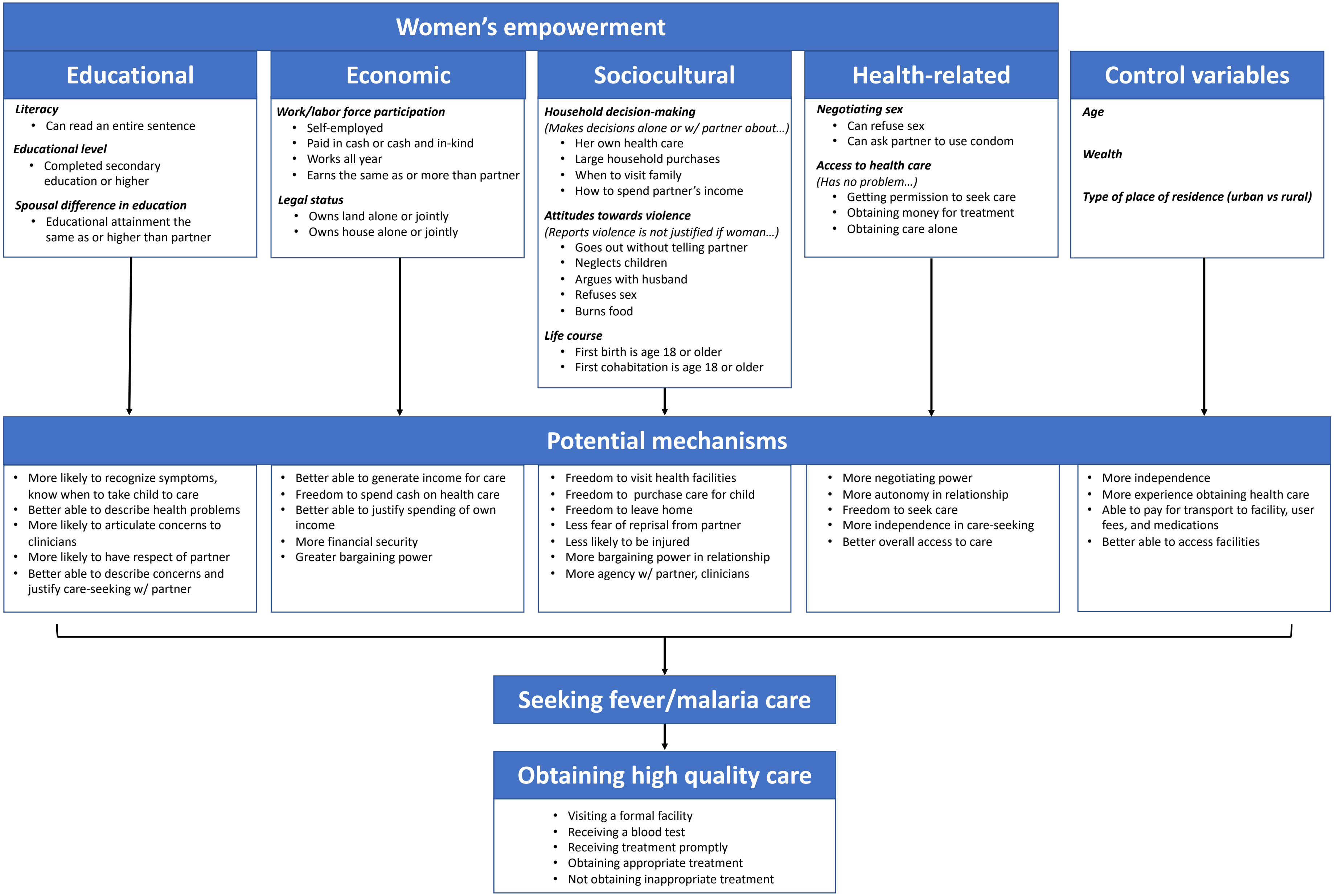

Table S1. Dimensions, domains, and indicators of women's empowerment in the Demographic and Health Surveys.

Dimension	Domain	Indicator	More empowered (code=1)	Less empowered (code=0)
**Educational empowerment**	Literacy	Literacy	Woman was able to read an entire sentence	Woman could not read or was able to read only parts of a sentence
	Educational attainment	Educational attainment	Woman completed secondary education	Woman completed no education or primary school only
	Spousal difference in education	Spousal difference in education	Woman had the same or higher educational attainment as her spouse/partner	Woman had lower educational attainment than her spouse/partner
**Economic empowerment**	Work/labor force participation	Type of employment	Woman was self-employed	Woman was unemployed or employed by someone else
		Type of earnings	Woman received cash or cash and in-kind earnings	Woman was unpaid or received in-kind earnings only
		Timing of employment	Woman was employed throughout the year	Woman was employed seasonally, occasionally, or on a temporary basis
		Relative income	Woman earned more or about the same as her spouse/partner	Woman made less than her spouse/partner or did not know
	Legal status	Land ownership	Woman owned land alone or jointly with her spouse/partner, or both	Woman did not own land
		Home ownership	Woman owned a home alone or jointly with her spouse/partner, or both	Woman did not own a home
**Sociocultural empowerment**	Household decision-making	Decisions about own health care	Woman reported making decisions alone or jointly with her partner	Woman reported that decisions were made by her partner or another person alone
		Decisions about large household purchases	Woman reported making decisions alone or jointly with her partner	Woman reported that decisions were made by her partner or another person alone
		Decisions about visiting family	Woman reported making decisions alone or jointly with her partner	Woman reported that decisions were made by her partner or another person alone
		Decisions about spending spouse's/partner's income	Woman reported making decisions alone or jointly with her partner	Woman reported that decisions were made by her partner or another person alone

	Attitude towards violence	Violence is justified if a woman goes out without telling her husband	Woman reported that domestic violence is not justified	Woman reported that violence is justified or that she doesn't know
		Violence is justified if a woman neglects her children	Woman reported that domestic violence is not justified	Woman reported that violence is justified or that she doesn't know
		Violence is justified if a woman argues with her husband	Woman reported that domestic violence is not justified	Woman reported that violence is justified or that she doesn't know
		Violence is justified if a woman refuses sex	Woman reported that domestic violence is not justified	Woman reported that violence is justified or that she doesn't know
		Violence is justified if a woman burns food	Woman reported that domestic violence is not justified	Woman reported that violence is justified or that she doesn't know
	Life course	Age at first birth	Woman was age 18 or older	Woman was younger than age 18
		Age at first cohabitation	Woman was age 18 or older	Woman was younger than age 18
**Health-related empowerment**	Ability to negotiate sex	Refusing sex	Woman could refuse sex	Woman could not refuse sex or didn't know
		Asking spouse/partner to use a condom	Woman could ask spouse/partner to use a condom	Woman could not ask spouse/partner to use a condom or didn't know
	Access to health care	Getting permission to seek care	Woman said this was not a big problem	Woman said this was a big problem
		Obtaining money for treatment	Woman said this was not a big problem	Woman said this was a big problem
		Obtaining care alone	Woman said this was not a big problem	Woman said this was a big problem

Figure S2. Proportion of children obtaining components of care for fever (N=25871) or malaria (N=4731) by country.*, †

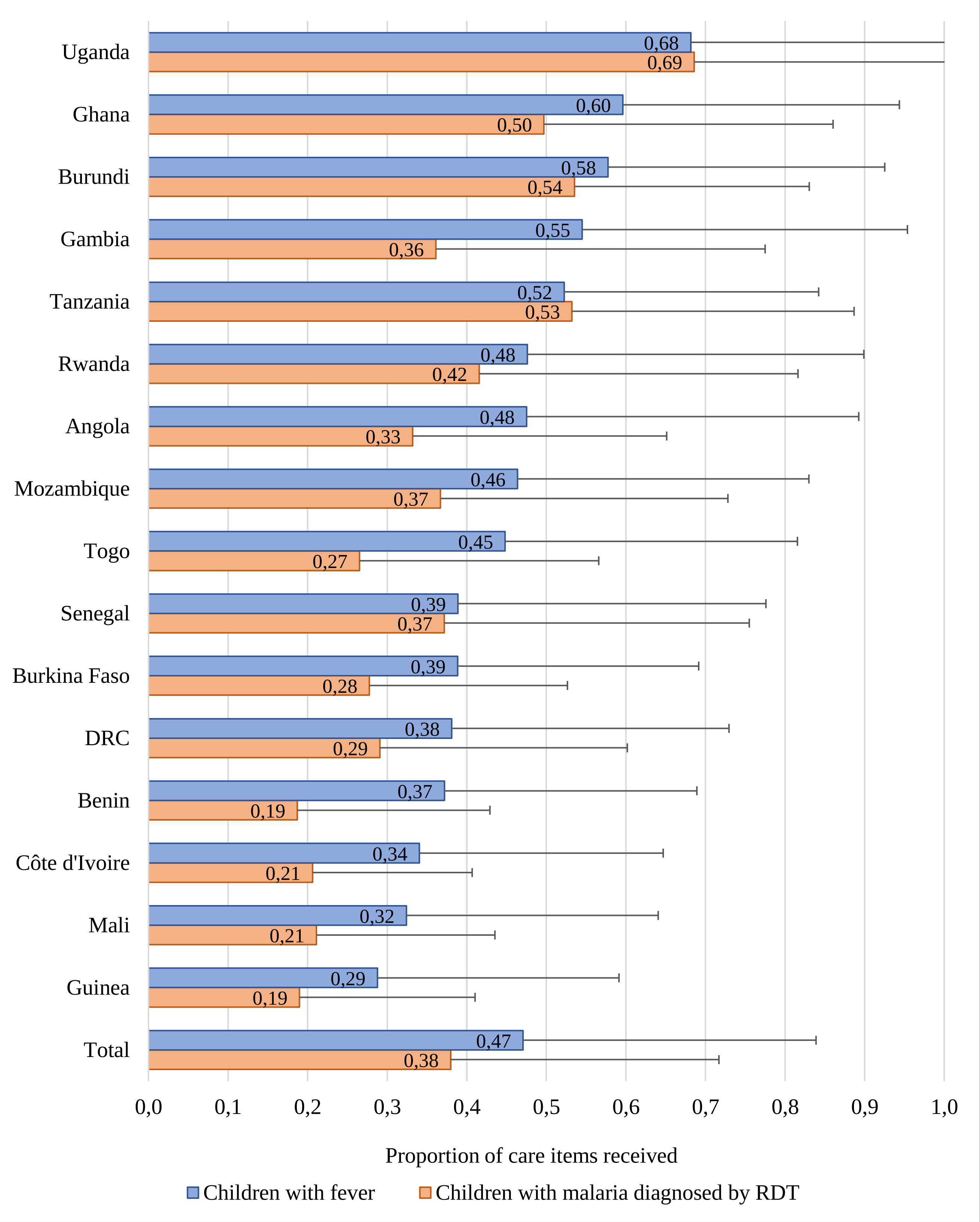

DRC – Democratic Republic of the Congo
*Care items for children with fever include seeking care at a formal facility, having blood taken, and not receiving inappropriate treatment. Care items for children with malaria include seeking care at a formal facility, having blood taken, beginning treatment the same or next day, receiving appropriate treatment, and not receiving inappropriate treatment.
†Whiskers indicate one standard deviation from the average proportion of care items received.

Table S2. Proportion of empowerment factors experienced by mothers of children with fever by country (N=25871)*

Country	Educational		Economic		Sociocultural		Health-related		All empowerment factors	
	Mean	(SD)	Mean	(SD)	Mean	(SD)	Mean	(SD)	Mean	(SD)
Angola	0.29	(0.36)	0.48	(0.30)	0.61	(0.23)	0.55	(0.31)	0.48	(0.18)
Benin	0.29	(0.29)	0.38	(0.27)	0.68	(0.21)	0.59	(0.29)	0.48	(0.15)
Burkina Faso	0.34	(0.21)	0.33	(0.28)	0.51	(0.21)	0.53	(0.27)	0.43	(0.13)
Burundi	0.43	(0.30)	0.71	(0.24)	0.66	(0.24)	0.65	(0.23)	0.61	(0.14)
Côte d'Ivoire	0.32	(0.29)	0.45	(0.30)	0.49	(0.24)	0.58	(0.28)	0.46	(0.15)
DRC	0.38	(0.40)	0.52	(0.29)	0.49	(0.24)	0.53	(0.27)	0.48	(0.16)
Gambia	0.42	(0.34)	0.34	(0.25)	0.51	(0.23)	0.67	(0.25)	0.48	(0.15)
Ghana	0.44	(0.35)	0.38	(0.27)	0.68	(0.22)	0.73	(0.24)	0.56	(0.16)
Guinea	0.31	(0.23)	0.39	(0.25)	0.33	(0.21)	0.36	(0.39)	0.35	(0.15)
Mali	0.35	(0.23)	0.51	(0.33)	0.39	(0.22)	0.44	(0.26)	0.42	(0.13)
Mozambique	0.34	(0.32)	0.61	(0.36)	0.64	(0.22)	0.66	(0.29)	0.56	(0.15)
Rwanda	0.51	(0.29)	0.62	(0.27)	0.75	(0.21)	0.76	(0.21)	0.66	(0.15)
Senegal	0.38	(0.31)	0.22	(0.24)	0.47	(0.27)	0.51	(0.25)	0.39	(0.16)
Tanzania	0.50	(0.34)	0.40	(0.28)	0.55	(0.25)	0.66	(0.32)	0.53	(0.17)
Togo	0.34	(0.35)	0.31	(0.21)	0.60	(0.22)	0.66	(0.26)	0.48	(0.15)
Uganda	0.38	(0.35)	0.51	(0.29)	0.57	(0.24)	0.75	(0.22)	0.55	(0.15)
**Total**	0.38	(0.32)	0.47	(0.31)	0.56	(0.25)	0.60	(0.29)	0.50	(0.17)

DRC – Democratic Republic of the Congo
*Educational empowerment is an average of the proportion of empowerment factors a woman experiences in three domains: literacy, educational level, and spousal difference in education (three indicators total). Economic empowerment is an average of the proportion of empowerment factors a woman experiences in two domains: work/labor force participation and legal status (six indicators total). Sociocultural empowerment is an average of the proportion of empowerment factors a woman experiences in three domains: household decision-making, attitudes towards violence, and life course (11 indicators total). Health-related empowerment is an average of the proportion of empowerment factors a woman experiences in two domains: negotiating sex and access to health care (five indicators total).

Table S3. Bivariable associations of empowerment with receipt of quality care among children with fever (N=25871) and malaria (N=4731).*, †, ‡

Care component	Educational empowerment		Economic empowerment		Sociocultural empowerment		Health-related empowerment	
**Children with fever in last two weeks (N=25871)**								
	Mean (SD)	*P*-value F test	Mean (SD)	*P*-value F test	Mean (SD)	*P*-value F test	Mean (SD)	*P*-value F test
Sought any treatment								
No	0.35 (0.30)	**<0.001**	0.45 (0.31)	0.187	0.53 (0.25)	**0.003**	0.56 (0.29)	**<0.001**
Yes	0.39 (0.33)		0.48 (0.31)		0.57 (0.25)		0.63 (0.28)	
Sought treatment at formal facility								
No	0.36 (0.32)	**<0.001**	0.45 (0.30)	0.092	0.53 (0.25)	**0.001**	0.57 (0.29)	**<0.001**
Yes	0.40 (0.33)		0.48 (0.31)		0.58 (0.25)		0.64 (0.28)	
Blood taken from finger/heel for testing								
No	0.36 (0.32)	**<0.001**	0.44 (0.30)	**0.035**	0.53 (0.25)	**<0.001**	0.57 (0.29)	**<0.001**
Yes	0.42 (0.34)		0.54 (0.31)		0.61 (0.25)		0.67 (0.26)	
Did not receive inappropriate treatment								
No	0.38 (0.32)	0.806	0.48 (0.31)	0.434	0.55 (0.25)	0.723	0.60 (0.29)	0.505
Yes	0.38 (0.33)		0.46 (0.31)		0.56 (0.25)		0.61 (0.28)	
**Children with malaria diagnosed by RDT (N=4731)**								
	Mean (SD)	*P*-value F test	Mean (SD)	*P*-value F test	Mean (SD)	*P*-value F test	Mean (SD)	*P*-value F test
Sought any treatment								
No	0.32 (0.26)	0.120	0.47 (0.30)	0.316	0.50 (0.25)	0.103	0.52 (0.29)	**0.006**
Yes	0.34 (0.29)		0.50 (0.31)		0.53 (0.24)		0.58 (0.28)	
Sought treatment at formal facility								
No	0.32 (0.27)	**0.003**	0.47 (0.30)	0.182	0.50 (0.24)	**0.024**	0.52 (0.29)	**0.001**
Yes	0.35 (0.29)		0.51 (0.31)		0.55 (0.25)		0.61 (0.28)	

Blood taken from finger/heel for testing								
No	0.31 (0.26)	**0.013**	0.45 (0.30)	0.077	0.49 (0.24)	**0.005**	0.52 (0.29)	**<0.001**
Yes	0.37 (0.31)		0.58 (0.30)		0.58 (0.24)		0.65 (0.26)	
Began treatment for malaria same or next day								
No	0.32 (0.27)	**0.002**	0.46 (0.30)	**0.021**	0.50 (0.24)	**0.002**	0.53 (0.29)	**<0.001**
Yes	0.36 (0.30)		0.56 (0.30)		0.57 (0.25)		0.64 (0.27)	
Received appropriate treatment								
No	0.32 (0.26)	**0.016**	0.48 (0.31)	0.380	0.51 (0.25)	0.075	0.54 (0.29)	**0.021**
Yes	0.36 (0.32)		0.52 (0.30)		0.55 (0.25)		0.64 (0.28)	
Did not receive inappropriate treatment								
No	0.33 (0.27)	0.882	0.48 (0.31)	0.632	0.51 (0.24)	0.316	0.55 (0.28)	0.638
Yes	0.33 (0.28)		0.50 (0.30)		0.53 (0.25)		0.57 (0.29)	

SD – standard deviation

*Educational empowerment is an average of the proportion of empowerment factors a woman experiences in three domains: literacy, educational level, and spousal difference in education (three indicators total). Economic empowerment is an average of the proportion of empowerment factors a woman experiences in two domains: work/labor force participation and legal status (six indicators total). Sociocultural empowerment is an average of the proportion of empowerment factors a woman experiences in three domains: household decision-making, attitudes towards violence, and life course (11 indicators total). Health-related empowerment is an average of the proportion of empowerment factors a woman experiences in two domains: negotiating sex and access to health care (five indicators total).

†"Sought treatment at formal facility" is defined as seeking care at a formal facility or provider, such as a government health center, rather than an informal provider such as a traditional practitioner or marketplace. "Received appropriate treatment" is defined as receipt of an antimalarial deemed appropriate by each country's national malaria treatment guidelines for either uncomplicated or severe malaria (typically an artemisinin-based combination therapy). "Did not receive inappropriate treatment" is defined as avoidance of a contraindicated medication or an unrecommended drug for a positive malaria diagnosis, a negative malaria diagnosis, or fever but unknown malaria according to country-specific guidelines.

‡Estimates were clustered at the country level. Bold values are statistically significant at the P=0.05 level.

Table S4. Multivariable hurdle regressions on care seeking and receipt of quality care for children with fever (N=25871) and malaria (N=4731) with overall empowerment index).*, †, ‡

	Children with fever in the last two weeks				Children with malaria diagnosed by RDT			
	Logistic part: Sought care (yes or no)		Poisson part: Number of care items (if care was sought)		Logistic part: Sought care (yes or no)		Poisson part: Number of care items (if care was sought)	
**Variable**	**AOR**	**95% CI**	**AIRR**	**95% CI**	**AOR**	**95% CI**	**AIRR**	**95% CI**
Overall empowerment	**2.01**	**(1.570 - 2.561)**	**1.13**	**(1.078 - 1.179)**	1.07	(0.588 - 1.949)	**1.29**	**(1.145 - 1.447)**
Female	1.01	(0.959 - 1.068)	0.99	(0.986 - 1.003)	0.97	(0.864 - 1.098)	0.99	(0.972 - 1.018)
Child age	1.01	(0.984 - 1.044)	1.00	(0.990 - 1.002)	**0.92**	**(0.866 - 0.979)**	1.01	(0.992 - 1.021)
Mother age	**0.99**	**(0.987 - 0.996)**	**0.99**	**(0.998 - 0.999)**	0.99	(0.981 - 1.003)	1.00	(0.998 - 1.002)
Rural	0.91	(0.795 - 1.052)	0.98	(0.953 - 1.010)	0.97	(0.735 - 1.279)	0.97	(0.912 - 1.022)
Wealth (ref: Poorest quintile)								
Second quintile	1.06	(0.926 - 1.205)	1.00	(0.966 - 1.037)	1.07	(0.939 - 1.224)	1.01	(0.953 - 1.067)
Third quintile	1.16	(0.978 - 1.368)	1.00	(0.963 - 1.044)	1.17	(0.895 - 1.536)	1.03	(0.974 - 1.083)
Fourth quintile	**1.25**	**(1.027 - 1.530)**	1.01	(0.961 - 1.051)	**1.36**	**(1.077 - 1.722)**	1.05	(0.975 - 1.128)
Richest quintile	**1.59**	**(1.182 - 2.143)**	1.01	(0.957 - 1.060)	**2.25**	**(1.667 - 3.037)**	**1.09**	**(1.000 - 1.178)**
Observations	25871		17756		4731		3225	

AOR – adjusted odds ratio, AIRR – adjusted incidence rate ratio, CI – confidence interval

*Overall empowerment is an average of four dimensions of empowerment factors: Educational, economic, sociocultural, and health-related. Educational empowerment is an average of the proportion of empowerment factors a woman experiences in three domains: literacy, educational level, and spousal difference in education (three indicators total). Economic empowerment is an average of the proportion of empowerment factors a woman experiences in two domains: work/labor force participation and legal status (six indicators total). Sociocultural empowerment is an average of the proportion of empowerment factors a woman experiences in three domains: household decision-making, attitudes towards violence, and life course (11 indicators total). Health-related empowerment is an average of the proportion of empowerment factors a woman experiences in two domains: negotiating sex and access to health care (five indicators total).

†Care items for children with fever include seeking care at a formal facility, having blood taken, and not receiving inappropriate treatment. Care items for children with malaria include seeking care at a formal facility, having blood taken, beginning treatment the same or next day, receiving appropriate treatment, and not receiving inappropriate treatment.

‡Estimates were obtained using Poisson hurdle regression clustered at the country level. All models included country fixed effects. Bold values are statistically significant at the P=0.05 level.

Table S5. Multivariable logistic regression models for each care activity for children with fever (N=25871).*, †, ‡

	Sought any treatment		Sought treatment at formal facility		Blood taken from finger/heel for testing		Did not receive inappropriate treatment	
**Variable**	**AOR**	**95% CI**	**AOR**	**95% CI**	**AOR**	**95% CI**	**AOR**	**95% CI**
Educational empowerment	**1.11**	**(1.037 - 1.187)**	1.09	(0.997 - 1.192)	**1.11**	**(1.004 - 1.236)**	0.98	(0.874 - 1.106)
Economic empowerment	1.14	(0.995 - 1.312)	**1.28**	**(1.055 - 1.563)**	**1.37**	**(1.141 - 1.633)**	0.88	(0.737 - 1.054)
Sociocultural empowerment	**1.22**	**(1.072 - 1.379)**	**1.34**	**(1.152 - 1.564)**	1.18	(0.969 - 1.446)	**1.24**	**(1.046 - 1.480)**
Health empowerment	**1.32**	**(1.079 - 1.621)**	**1.44**	**(1.260 - 1.653)**	**1.45**	**(1.172 - 1.798)**	0.91	(0.785 - 1.051)
Female	1.01	(0.958 - 1.068)	0.98	(0.931 - 1.023)	0.96	(0.918 - 1.009)	1.00	(0.936 - 1.066)
Child age	1.01	(0.985 - 1.045)	0.98	(0.951 - 1.012)	**1.09**	**(1.064 - 1.125)**	**0.89**	**(0.863 - 0.926)**
Mother age	**0.99**	**(0.987 - 0.996)**	**0.99**	**(0.989 - 0.995)**	**0.99**	**(0.983 - 0.995)**	1.00	(0.994 - 1.011)
Rural	0.92	(0.799 - 1.050)	0.92	(0.777 - 1.090)	**0.77**	**(0.645 - 0.929)**	1.04	(0.898 - 1.215)
Wealth (ref: Poorest quintile)								
Second quintile	1.06	(0.925 - 1.204)	1.06	(0.920 - 1.230)	0.98	(0.821 - 1.164)	0.95	(0.849 - 1.055)
Third quintile	1.15	(0.975 - 1.363)	1.14	(0.914 - 1.426)	1.01	(0.783 - 1.292)	0.94	(0.803 - 1.095)
Fourth quintile	**1.25**	**(1.023 - 1.520)**	1.22	(0.926 - 1.605)	1.01	(0.760 - 1.337)	0.89	(0.687 - 1.154)
Richest quintile	**1.58**	**(1.179 - 2.129)**	1.39	(0.964 - 1.992)	1.14	(0.721 - 1.809)	0.84	(0.587 - 1.199)
Observations	25871		25871		25871		25871	

AOR – adjusted odds ratio, CI – confidence interval

*Educational empowerment is an average of the proportion of empowerment factors a woman experiences in three domains: literacy, educational level, and spousal difference in education (three indicators total). Economic empowerment is an average of the proportion of empowerment factors a woman experiences in two domains: work/labor force participation and legal status (six indicators total). Sociocultural empowerment is an average of the proportion of empowerment factors a woman experiences in three domains: household decision-making, attitudes towards violence, and life course (11 indicators total). Health-related empowerment is an average of the proportion of empowerment factors a woman experiences in two domains: negotiating sex and access to health care (five indicators total).

†“Sought treatment at formal facility” is defined as seeking care at a formal facility or provider, such as a government health center, rather than an informal provider such as a traditional practitioner or marketplace. “Did not receive inappropriate treatment” is defined as avoidance of a contraindicated medication or an unrecommended drug for a positive malaria diagnosis, a negative malaria diagnosis, or fever but unknown malaria according to country-specific guidelines.

‡Estimates were obtained using logistic regression clustered at the country level. All models included country fixed effects. Bold values are statistically significant at the P=0.05 level.

Table S6. Multivariable logistic regression models for each care item for children with malaria diagnosed by RDT (N=4731).*, †, ‡

	Sought any treatment		Sought treatment at formal facility		Blood taken from finger/heel for testing		Began treatment for malaria same or next day		Received appropriate treatment		Did not receive inappropriate treatment	
**Variable**	**AOR**	**95% CI**	**AOR**	**95% CI**	**AOR**	**95% CI**	**AOR**	**95% CI**	**AOR**	**95% CI**	**AOR**	**95% CI**
Educational empowerment	0.90	(0.737 - 1.105)	1.02	(0.948 - 1.104)	1.22	(0.979 - 1.516)	1.03	(0.786 - 1.360)	1.17	(0.895 - 1.530)	1.06	(0.845 - 1.331)
Economic empowerment	1.03	(0.726 - 1.448)	**1.31**	**(1.020 - 1.671)**	0.90	(0.559 - 1.459)	**1.43**	**(1.026 - 2.001)**	1.17	(0.776 - 1.765)	0.90	(0.640 - 1.262)
Sociocultural empowerment	1.00	(0.810 - 1.243)	1.30	(0.968 - 1.733)	1.22	(0.849 - 1.740)	**1.37**	**(1.058 - 1.771)**	1.32	(0.908 - 1.927)	**1.33**	**(1.023 - 1.737)**
Health-related empowerment	1.14	(0.895 - 1.454)	**1.55**	**(1.179 - 2.040)**	**1.68**	**(1.086 - 2.589)**	**1.55**	**(1.162 - 2.061)**	1.19	(0.799 - 1.770)	**0.83**	**(0.716 - 0.973)**
Female	0.97	(0.863 - 1.099)	0.95	(0.851 - 1.071)	1.06	(0.970 - 1.168)	0.99	(0.877 - 1.120)	0.94	(0.845 - 1.048)	1.02	(0.954 - 1.087)
Child age	**0.92**	**(0.865 - 0.979)**	0.93	(0.841 - 1.034)	0.95	(0.890 - 1.004)	1.05	(0.982 - 1.116)	1.02	(0.924 - 1.117)	1.00	(0.964 - 1.041)
Mother age	0.99	(0.982 - 1.002)	**0.99**	**(0.975 - 0.998)**	1.00	(0.985 - 1.011)	1.00	(0.988 - 1.008)	1.00	(0.993 - 1.011)	1.01	(0.996 - 1.022)
Rural	0.96	(0.738 - 1.261)	0.88	(0.637 - 1.224)	**0.80**	**(0.657 - 0.967)**	0.93	(0.785 - 1.108)	0.87	(0.645 - 1.169)	1.05	(0.862 - 1.280)
Wealth (ref: Poorest quintile)												
Second quintile	1.07	(0.937 - 1.217)	1.03	(0.852 - 1.235)	1.18	(0.989 - 1.414)	0.98	(0.803 - 1.186)	1.07	(0.864 - 1.333)	0.91	(0.741 - 1.110)
Third quintile	1.17	(0.893 - 1.528)	1.13	(0.865 - 1.480)	1.13	(0.763 - 1.666)	1.14	(0.851 - 1.522)	1.14	(0.824 - 1.575)	0.91	(0.744 - 1.106)
Fourth quintile	**1.36**	**(1.068 - 1.729)**	1.26	(0.959 - 1.656)	**1.45**	**(1.137 - 1.856)**	1.27	(0.812 - 1.980)	**1.49**	**(1.114 - 2.002)**	0.92	(0.650 - 1.293)
Richest quintile	**2.26**	**(1.646 - 3.099)**	**1.67**	**(1.046 - 2.655)**	**1.84**	**(1.268 - 2.680)**	1.70	(0.963 - 2.996)	**2.28**	**(1.758 - 2.950)**	0.75	(0.508 - 1.111)
Observations	4731		4731		4731		4731		4731		4731	

AOR – adjusted odds ratio, CI – confidence interval
*Educational empowerment is an average of the proportion of empowerment factors a woman experiences in three domains: literacy, educational level, and spousal difference in education (three indicators total). Economic empowerment is an average of the proportion of empowerment factors a woman experiences in two domains: work/labor force participation and legal status (six indicators total). Sociocultural empowerment is an average of the proportion of empowerment factors a woman experiences in three domains: household decision-making, attitudes towards violence, and life course (11 indicators total). Health-related empowerment is an average of the proportion of empowerment factors a woman experiences in two domains: negotiating sex and access to health care (five indicators total).
†"Sought treatment at formal facility" is defined as seeking care at a formal facility or provider, such as a government health center, rather than an informal provider such as a traditional practitioner or marketplace. "Received appropriate treatment" is defined as receipt of an antimalarial deemed appropriate by each country's national malaria treatment guidelines for either uncomplicated or severe malaria (typically an artemisinin-based combination therapy). "Did not receive inappropriate treatment" is defined as avoidance of a contraindicated medication or an unrecommended drug for a positive malaria diagnosis, a negative malaria diagnosis, or fever but unknown malaria according to country-specific guidelines.
‡Estimates were obtained using logistic regression clustered at the country level. All models included country fixed effects. Bold values are statistically significant at the P=0.05 level.

Table S7. Multivariable zero-inflated Poisson regression models of care seeking and receipt of quality care for fever (N=25871) and malaria (N=4731).*, †, ‡

	Children with fever in the last two weeks				Children with malaria diagnosed by RDT			
	Zero inflated part: Sought care (yes or no)		Poisson part: Number of care items (if care was sought)		Zero inflated part: Sought care (yes or no)		Poisson part: Number of care items (if care was sought)	
Variable	AOR	95% CI	AIRR	95% CI	AOR	95% CI	AIRR	95% CI
Educational empowerment	**0.89**	**(0.814 - 0.973)**	1.01	(0.990 - 1.039)	1.15	(0.887 - 1.504)	1.04	(0.979 - 1.096)
Economic empowerment	0.87	(0.729 - 1.041)	1.04	(0.998 - 1.085)	0.96	(0.656 - 1.418)	1.05	(0.972 - 1.143)
Sociocultural empowerment	**0.83**	**(0.714 - 0.953)**	**1.06**	**(1.020 - 1.102)**	1.13	(0.900 - 1.415)	**1.12**	**(1.027 - 1.223)**
Health-related empowerment	**0.72**	**(0.542 - 0.963)**	**1.05**	**(1.002 - 1.104)**	0.89	(0.659 - 1.212)	**1.10**	**(1.024 - 1.189)**
Female	0.98	(0.915 - 1.042)	0.99	(0.982 - 1.004)	1.02	(0.876 - 1.186)	0.99	(0.965 - 1.019)
Child age	0.97	(0.937 - 1.010)	0.99	(0.985 - 1.003)	**1.11**	**(1.033 - 1.184)**	1.01	(0.991 - 1.025)
Mother age	**1.01**	**(1.004 - 1.016)**	**1.00**	**(0.998 - 1.000)**	1.01	(0.998 - 1.021)	1.00	(0.998 - 1.002)
Rural	1.09	(0.909 - 1.299)	0.97	(0.938 - 1.012)	1.04	(0.704 - 1.525)	0.96	(0.892 - 1.037)
Wealth (ref: Poorest quintile)								
Second quintile	0.93	(0.782 - 1.103)	1.00	(0.958 - 1.042)	0.94	(0.790 - 1.108)	1.01	(0.944 - 1.076)
Third quintile	0.84	(0.680 - 1.032)	1.00	(0.955 - 1.053)	0.87	(0.646 - 1.181)	1.03	(0.971 - 1.100)
Fourth quintile	**0.75**	**(0.581 - 0.956)**	1.00	(0.949 - 1.059)	**0.70**	**(0.525 - 0.940)**	1.05	(0.966 - 1.143)
Richest quintile	**0.54**	**(0.370 - 0.798)**	1.01	(0.951 - 1.074)	**0.42**	**(0.276 - 0.649)**	1.11	(0.996 - 1.240)

AOR – adjusted odds ratio, AIRR – adjusted incidence rate ratio, CI – confidence interval

*Educational empowerment is an average of the proportion of empowerment factors a woman experiences in three domains: literacy, educational level, and spousal difference in education (three indicators total). Economic empowerment is an average of the proportion of empowerment factors a woman experiences in two domains: work/labor force participation and legal status (six indicators total). Sociocultural empowerment is an average of the proportion of empowerment factors a woman experiences in three domains: household decision-making, attitudes towards violence, and life course (11 indicators total). Health-related empowerment is an average of the proportion of empowerment factors a woman experiences in two domains: negotiating sex and access to health care (five indicators total).

†Care items for children with fever include seeking care at a formal facility, having blood taken, and not receiving inappropriate treatment. Care items for children with malaria include seeking care at a formal facility, having blood taken, beginning treatment the same or next day, receiving appropriate treatment, and not receiving inappropriate treatment.

‡Estimates were obtained using zero-inflated Poisson regression clustered at the country level. All models included country fixed effects. Bold values are statistically significant at the P=0.05 level.
